# Neuronal-Plasticity and Reward-Propagation Improved Recurrent Spiking Neural Networks

**DOI:** 10.3389/fnins.2021.654786

**Published:** 2021-03-12

**Authors:** Shuncheng Jia, Tielin Zhang, Xiang Cheng, Hongxing Liu, Bo Xu

**Affiliations:** ^1^Research Center for Brain-Inspired Intelligence, Institute of Automation, Chinese Academy of Sciences (CASIA), Beijing, China; ^2^School of Artificial Intelligence, University of Chinese Academy of Sciences (UCAS), Beijing, China; ^3^Faculty of Information Technology, Beijing University of Technology, Beijing, China; ^4^Center for Excellence in Brain Science and Intelligence Technology, Chinese Academy of Sciences, Shanghai, China

**Keywords:** spiking neural network, neuronal plasticity, synaptic plasticity, reward propagation, sparse connections

## Abstract

Different types of dynamics and plasticity principles found through natural neural networks have been well-applied on Spiking neural networks (SNNs) because of their biologically-plausible efficient and robust computations compared to their counterpart deep neural networks (DNNs). Here, we further propose a special Neuronal-plasticity and Reward-propagation improved Recurrent SNN (NRR-SNN). The historically-related adaptive threshold with two channels is highlighted as important neuronal plasticity for increasing the neuronal dynamics, and then global labels instead of errors are used as a reward for the paralleling gradient propagation. Besides, a recurrent loop with proper sparseness is designed for robust computation. Higher accuracy and stronger robust computation are achieved on two sequential datasets (i.e., TIDigits and TIMIT datasets), which to some extent, shows the power of the proposed NRR-SNN with biologically-plausible improvements.

## 1. Introduction

Many different types of deep neural networks (DNNs) have been proposed for efficient machine learning on image classification (Ciregan et al., 2012), recognition (Nguyen et al., 2015), memory association (He et al., 2017), and prediction (Kim et al., 2017). However, with the rapid development of DNNs, there are some shortcomings hindering their advance.

- The first problem is the increasing number of synaptic parameters. Different types of structures instead of neurons play important roles in different functions of DNNs, where nearly all artificial neurons use a Sigmoid-like activation function for simple non-linear input-output mapping. The unbalanced complexity between artificial neurons and networks allows DNNs to contain a large number of network parameters that can be tuned.
- The second problem is the slow backpropagation (BP) with a high computational cost, which is also considered to be not biologically-plausible. In DNNs, the BP interleaves with feedforward propagation sequentially, and the error signals have to be backpropagated from the output neurons to hidden neurons layer-by-layer, with a risk of gradient disappearance or gradient explosion, especially for extremely-deep networks. The nature of supervised and synchronous computation of DNNs also makes them difficult to accelerate with parallel computation.
- The third problem is that all of the artificial neurons in DNNs during the BP procedure have to satisfy the limitation of mathematical differentiability, which obviously lacks support from biological verification, where the non-differential spike-type signals are everywhere, caused by the time slot of membrane potential at firing threshold, the probabilistic firing of a specific spike, or the hard refractory time for stop firing.
- The fourth problem is the separation of spatial and temporal information with different network architectures. For example, the convolutional kernels are carefully designed for efficient spatial information integration, and the recurrent loops (sparse or dense types) are successfully introduced for effective sequential information prediction, instead of simultaneous spatially-temporal information processing in biological networks.

Unlike DNNs, some other networks are designed to contain both biologically-realistic network structures and biologically-plausible tuning methods. A spiking neural network (SNN) is one of them, which contains spiking neurons with dynamic membrane potential and also dynamic synapses for spatially-temporal information processing. There are many advantages of SNNs compared to their counterpart DNNs. For example, the two-bit efficient encoding of information at the neuronal scale; the balanced complexity between the neuronal and network scales, i.e., with proper-sparseness connections (neurons only connect in a certain area) and far-more complex neurons (neurons with dynamic somas and dendrites).

Furthermore, SNNs prefer using the biologically-plausible tuning methods, such as spike-timing-dependent plasticity (STDP) (Dan and Poo, 2004), short-term plasticity (STP) (Zucker, 1989), pre-post membrane balanced plasticity (Zhang et al., 2018a,b), and excitatory-inhibitory balanced plasticity (Zeng et al., 2017). The long-term depression (LTD) (Ito, 1989) shows that the repeated low-frequency activation into postsynaptic neurons will reduce the transmission efficiency of synapses, while those with repeated high-frequency [long-term potentiation, LTP (Teyler and DiScenna, 1987)] will lead to synaptic enhancement. STDP (Bengio et al., 2017) shows that presynaptic and postsynaptic activations of different neurons in chronological order would result in different (with an increment or decrement) synaptic changes, i.e., if the postsynaptic neuron fired within 20 ms after the activation of the presynaptic neuron, it would cause LTP, or LTD. Additionally, more effective plasticity propagation rules have been elucidated and are well-applied in the training of SNNs. The reward propagation (Zhang et al., 2020b) describes an efficient label-based, instead of error-based, gradient propagation. Synaptic plasticity propagation describes LTP/LTD propagation in neighborhood synapses (Bi and Poo, 2001). Most of these plasticity propagation rules are biologically-plausible for the efficient learning of SNNs.

There are also some shortcomings of SNNs. First, due to the non-differential character of biological neurons in SNNs, the gradient backpropagation that is powered by tuning DNNs is not directly applicable on the training of SNNs; Second, ordinary SNNs have limited neuronal dynamics, omitting dynamic thresholds and other related features of biological networks. These phenomena make the current SNNs more closed to DNNs with an unbalanced complexity between local neurons and global networks, instead of a balanced complexity in biological networks.

This paper focuses more on the research on neuronal dynamics, learning plasticity, and sparseness architectures of SNNs, looking toward a more efficient biologically-plausible computation. Hence, under these considerations, the Neuronal-plasticity and Reward-propagation improved Recurrent SNN (NRR-SNN) is proposed for efficient and robust computations. The contribution of this paper can be concluded as follows:

- First, the historically-related two-channel adaptive threshold is highlighted as an important neuronal plasticity for increasing neuronal dynamics. This additional neuronal dynamic will integrate well with other dynamic membrane potentials (e.g., the leaky integrated-and-fire, LIF) for a stronger temporal information computation.
- Second, the global labels, instead of errors, are used as a reward for the gradient propagation. This new learning method can also be parallelly computed to save on computational costs.
- Third, dynamic neurons are then connected in a recurrent loop with defined sparseness for the robust computation. Moreover, an additional parameter is set to represent the degree of sparseness to analyze the proposed algorithm's anti-noise performance.

The paper is organized as follows: The section 2 provides a brief introduction of related works. In section 3, some basic background knowledge about dynamic neurons, the procedure of feedforward propagation, and plasticity propagation in standard SNNs is provided. A detailed description of the proposed NRR-SNN is given in section 4, including the dynamic nodes with neuronal plasticity, the architecture with different sparseness, and the tuning method reward propagation. Section 5 details the proposed algorithm's performance on two standard sequential datasets (i.e., TIDigits and TIMIT) on their efficient and robust computations. Further discussions and conclusions will be provided in the section 6.

## 2. Related Works

The multi-scale plasticity in SNN covers the neuronal plasticity, synaptic plasticity, and plasticity propagations. Neuronal plasticity plays a critical role in the dynamic information processing of the biological neural network (Hassabis et al., 2017; Zhang et al., 2020a). The standard neurons in SNNs include the H-H model (Hodgkin and Huxley, 1939, 1945; Noble, 1962), LIF model (Gerstner et al., 2014), SRM model (Gerstner et al., 1993; Gerstner, 2008), and Izhikevich model (Izhikevich, 2003). The VPSNN (short for voltage-dependent and plasticity-centric SNN) has been proposed, which contains the neuronal plasticity and focuses more on membrane potential dynamics with a static firing threshold (Zhang et al., 2018a). Yu et al. (2018) have also proposed several plasticity algorithms to deal with spike coding's neuronal plasticity during training.

Synaptic plasticity refers to the dynamic changes of synapses according to different tasks. Zenke et al. (Zenke and Ganguli, 2018) have proposed the SuperSpike, where a non-linear voltage-based three-factor learning rule was used to dynamically update neuronal plasticity at the synapse scale. Kheradpisheh et al. (2018) have proved that the STDP plasticity was simpler and superior to other unsupervised learning rules in the same network architectures.

The propagation of synaptic plasticity is closely related to the credit assignment of error signals in SNNs. Zhang et al. have given an overview introduction of several target propagation methods, such as error propagation, symbol propagation, and label propagation (Frenkel et al., 2019), where the reward propagation can propagate the reward (instead of the traditional error signals) directly to all hidden layers (instead of the traditional layer-to-layer backpropagation). This plasticity is biologically-plausible and will also be used as the main credit assignment of SNNs in our NRR-SNN algorithm. Zhao et al. (2020) have proposed a similar method, where global random feedback alignment is combined with STDP for efficient credit assignment.

Besides the plasticity, network structures have played important roles in network learning. Currently, the network structures in SNNs are similar to their counterpart DNNs (Lee et al., 2016; Wu et al., 2020a), depending on the requirement of different spatial or temporal tasks. For example, feedforward-type architectures are usually used during the spatial information processing (e.g., the image classification on the MNIST dataset) (Diehl and Cook, 2015; Zhang et al., 2020a), and recurrent-type architectures are constructed more for sequential information processing (e.g., the auditory sequence recognition on the TIDigits dataset) (Dong et al., 2018; Pan et al., 2019).

## 3. Background

### 3.1. Dynamic Spiking Neurons

**Figure 1:**
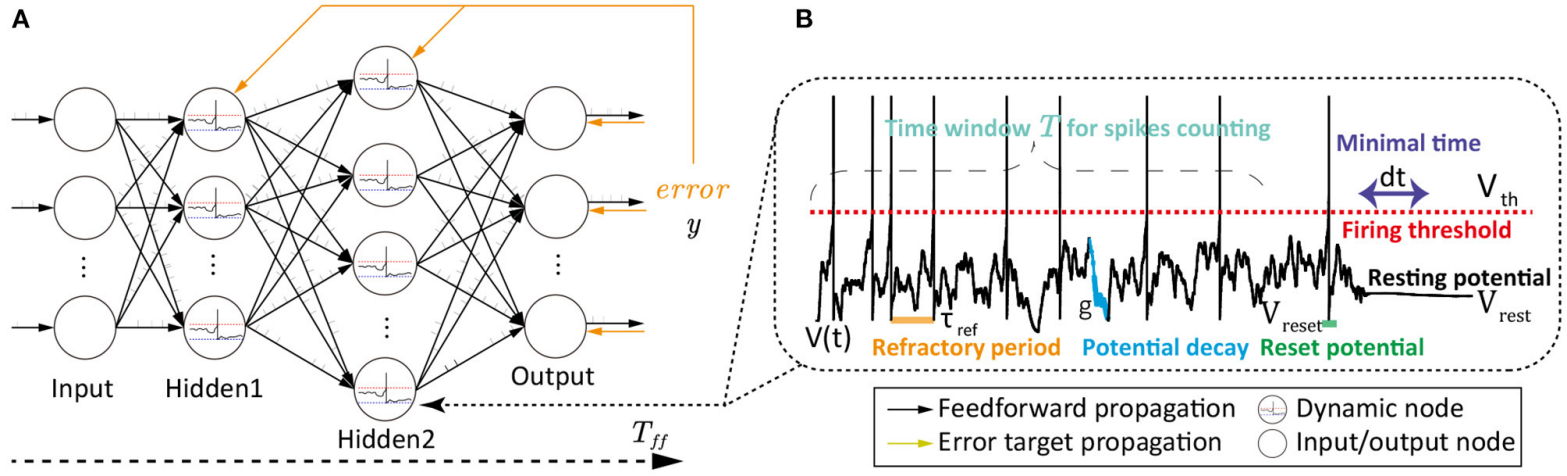
A schematic diagram depicting the SNN with dynamic neurons, feedforward spike propagation, and feedback error propagation. **(A)** The feedforward propagation and error target propagation in the standard SNN, containing dynamic neurons at spiking or resting states. **(B)** The dynamic LIF neuron with spikes and subthreshold membrane potential.

The dynamic spiking neurons in SNNs are not continuous in the real number field, which is different from the artificial neurons such as the Sigmoid activation function, Tanh activation function, and Rectified linear unit (ReLU). The standard LIF neuron is shown as follows:

(1) $$\left\{ \begin{array}{l} C\frac{dV_{i}(t)}{dt}=g(V_{i}(t)-V_{rest})+\sum_{j=1}^{N}W_{i,j}X_{j}(t)\\ \;V_{i}(t)=V_{reset}\;if(V_{i}(t)=V_{th},t-t_{spike}>\tau_{ref}) \end{array}\right.,$$

where *V*_*i*_(*t*) is membrane potential, *V*_*th*_ is firing threshold, *V*_*reset*_ is reset membrane potential (also generating a spike at the same time), and *V*_*rest*_ is the resting potential. *τ*_*ref*_ is the refractory time period, where the *V*_*i*_(*t*) will not increase toward the *V*_*th*_ at time *t* only if it is still during the period of *τ*_*ref*_. *X*_*j*_(*t*) is the receiving LIF neuron input from the presynaptic neuron *j*. One schematic diagram of dynamic LIF neuron is shown in Figure 1B.

### 3.2. Feedforward Propagation in SNN

Figure 1A shows the sequential spike trains in the feedforward propagation (labeled as period *T*_*ff*_) of SNNs for each epoch. For example, as a speech, it is spitted as *N* frames, and each frame is encoded as a normally-distributed spike train. Then these spike trains are sequentially inputted into the feedforward procedure of SNN. The information encoding in each LIF neuron with spikes is shown as follows:

(2) $$\{\begin{array}{l} \begin{array}{l} C\frac{dV_{i}^{f}(t)}{dt}=g(V_{i}(t)-V_{rest})(1-S^{f})+\sum_{j=1}^{N}W_{\;i,j}^{f}X_{j}(t) \end{array}\\ V_{i}^{f}(t)=V_{reset},S^{f}=1\;if\left(V_{i}^{f}(t)=V_{th}\right)\\ \begin{array}{ll} S^{f}=1 & if\left(t-t_{spike^{f}}<\tau_{ref},t\in \left(1,T_{1}\right)\right) \end{array} \end{array},$$

where $V_{i}^{f}\left(t\right)$ is the feedforward membrane potential with historically integrated states, *S* is a spike flag for the neuron, which indicates the number of spikes when the *V*_*i*_(*t*) (where $V_{i}^{f}\left(t\right)$ is part of *V*_*i*_(*t*)) reaches *V*_*th*_. The *S* also controls the refractory time period *τ*_*ref*_ by resetting the historical membrane potential *g*(*V*_*i*_(*t*) − *V*_*rest*_) instead of blocking the $V_{i}^{f}\left(t\right)$ directly.

### 3.3. Standard Target Propagation

The standard backpropagation (BP) (Rumelhart et al., 1986) uses the gradient descent algorithm to modify the synaptic weights layer-by-layer with the differential chain rule. However, the derivative of activation functions is usually less than 1, causing the backpropagated gradient to vanish in some deeper layers.

This study aims modify all synaptic weights parallelly without worrying about the gradient vanishing problem, especially for dynamic LIF neurons. Hence, we will pay more attention to the target propagation (Frenkel et al., 2019), as shown in Figure 1A, where the error or other reward-like signals are directly propagated from the output layer to all hidden layers parallelly without losing accuracy.

## 4. Method

**Figure 2:**
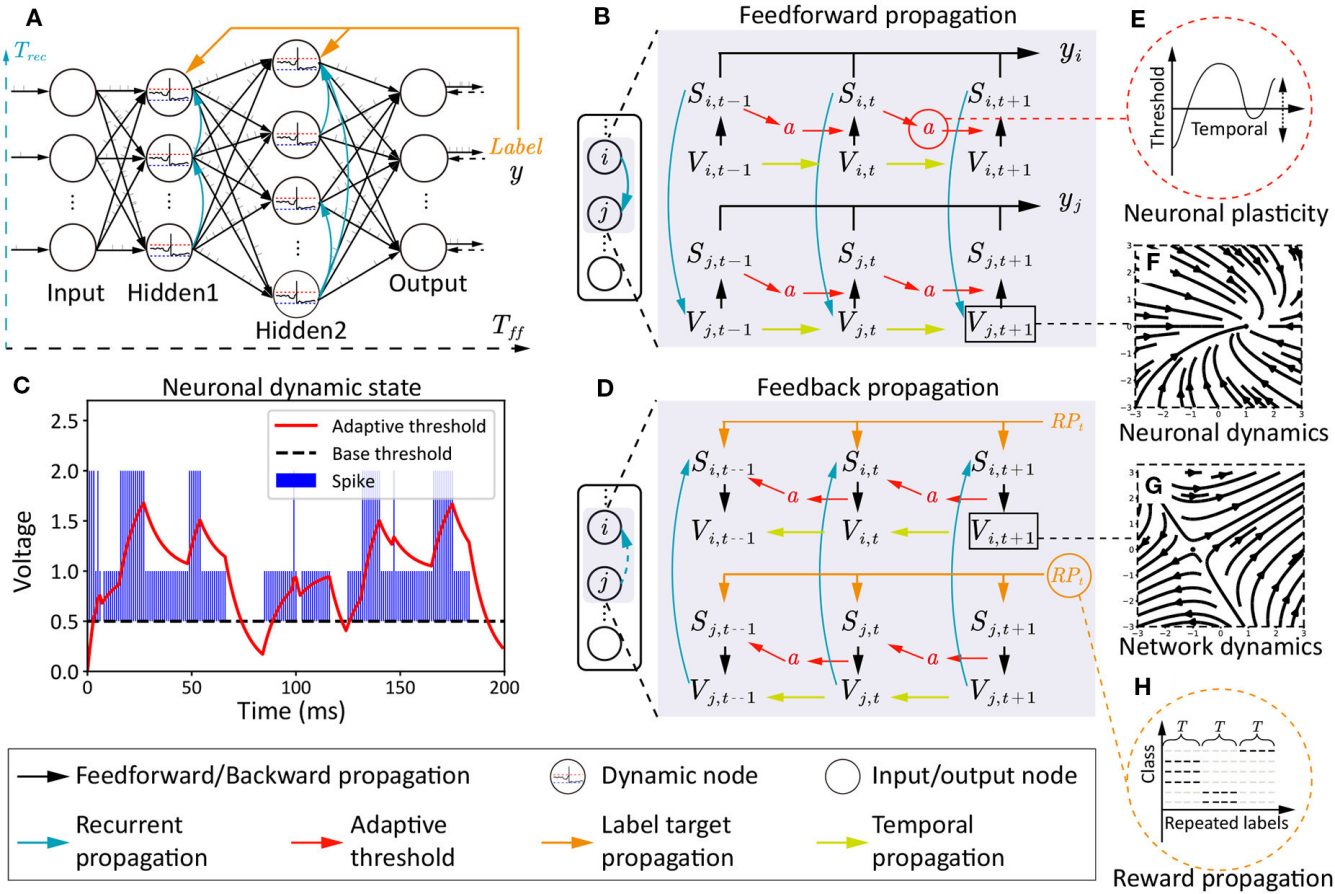
The architecture, two phases of information propagations, and multi-scale dynamics in NRR-SNN. **(A)** The SNN architecture with the feedforward period *T*_*ff*_, the recurrent period *T*_*rec*_, and the reward propagation with labels. **(B)** The feedforward information propagation from input neurons *V*_*i,t*_ to network output *y*_*j,t*_. **(C,E)** The two-channel neuronal plasticity related to spike trains. **(D)** The feedback information propagation from label *RP*_*t*_ to hidden neurons *V*_*i,t*_, *V*_*i,t*−*k*_ where *k* ∈ *T*. **(F,G)** The vector field examples of dynamic membrane potentials. **(H)** A diagram depicting the reward propagation with teaching signals of repeated labels.

Here, we will provide a detailed introduction about NRR-SNN, including three main parts: the neuronal plasticity with a 2-channel dynamic firing threshold; the recurrent connections with different proportions of sparseness; the reward propagation with the direct tuning of synaptic weights with loaded labels, as shown in Figure 2.

### 4.1. Neuronal Plasticity

The neuronal plasticity is different from traditional synaptic plasticity, where more dynamic characteristics within neurons are discussed for better spatially-temporal information processing. Here, we set an adaptive threshold with an ordinary differential equation (ODE). This is an ingenious effort to obtain a dynamic firing threshold with an attractor in ODE, instead of directly setting that as a predefined static value, as shown in the following equation:

(3) $$\begin{array}{r} \frac{da_{i}\left(t\right)}{dt}=\left(\alpha -1\right)a_{i}\left(t\right)+\beta \left(S^{f}+S^{r}\right), \end{array}$$

where *a*_*i*_(*t*) is a dynamic threshold with an equilibrium point of 0 without input spikes, or with another equilibrium point of $-\frac{\beta }{\alpha -1}$ given input spikes *S*^*f*^ + *S*^*r*^ from feedforward and recurrent channels. Hence, the ODE of membrane potential for LIF neuron is updated as follows:

(4) $$\begin{array}{r} C\frac{dV_{i}\left(t\right)}{dt}=g\left(V_{i}\left(t\right)-V_{rest}\right)\left(1-S^{f}-S^{r}\right)+\overset{N}{\underset{j=1}{\sum }}W_{i,j}X_{j}\left(t\right)-\gamma a_{i}\left(t\right), \end{array}$$

where during the period from the resetting to the firing of membrane potential, the dynamic threshold parameter *a*_*i*_(*t*) is accumulated gradually and eventually reached a relatively stable value. Because of the −*γa*_*i*_(*t*), the firing threshold is increased into *V*_*th*_ + *γa*_*i*_(*t*). For the *a*_*i*_(*t*), we can solve the stable value $a_{i}^{*}=\frac{\beta }{1-\alpha }\left(S^{f}+S^{r}\right)$.

In this paper, we provide *α*=0.9, *β*=0.1, and *γ*=1, therefore the stable a^*^=0 for no spikes, a^*^=1 for one spike, and a^*^=2 for spikes from two channels (i.e., the feedforward and recurrent channels). When $a_{i}\left(t\right)<\left(S^{f}+S^{r}\right)$, *a*_*i*_(*t*) will increase and the threshold will increase, otherwise, they will both decrease. It can be considered that the threshold will be changed dynamically with neurons' discharge. The adaptive threshold will also be increased or decreased when the firing frequency is higher or lower. Here, we use it as the main controlling part of neuronal plasticity.

### 4.2. Architecture With Sparse Loops

Recurrent connections show the dynamics at the network scale, as shown in Figure 2A, where neurons are connected within the inner hidden layers with defined or learnable connections. Hence, two types of membrane potentials are combined in the dynamic neurons. One is the recurrent membrane potential $V_{i}^{r}\left(t\right)$, and the other is the feedforward membrane potential $V_{i}^{f}\left(t\right)$. The definitions of these two types of membrane potential can be considered as two channels with the following equations:

(5) $$\left\{ \begin{array}{l} V_{i}^{f}(t)=V_{reset},S^{f}=1\;if(V_{i}^{f}(t)=V_{th})\\ \;V_{i}^{r}(t)\;=V_{reset},S^{r}=1\;if(V_{i}^{r}(t)=V_{th})\\ S^{f}=1\;if(t-t_{s^{f}}<\tau_{ref},t\in (1,T_{1}))\\ S^{r}=1\;if(t-t_{s^{r}}<\tau_{ref},t\in (1,T_{2})) \end{array}\right.,$$

where two spike flags (*S*^*f*^ and *S*^*r*^) are defined separately. The dynamic membrane potential of $V_{i}^{r}\left(t\right)$ and $V_{i}^{f}\left(t\right)$ are then integrated together, and defined as follows:

(6) $$\{\begin{array}{l} \begin{array}{l} C\frac{dV_{i}^{f}(t)}{dt}=g(V_{i}(t)-V_{rest})(1-S)+ \end{array}\sum_{j=1}^{N}W_{\;i,j}^{f}X_{j}(t)\\ \begin{array}{l} C\frac{dV_{i}^{r}(t)}{dt}=\sum_{j=1}^{N}W_{\;i,j}^{f}S \end{array}\\ \begin{array}{l} V_{i}(t)=V_{i}^{f}(t)+V_{i}^{r}(t) \end{array}\\ \begin{array}{l} S=S^{f}+S^{r} \end{array} \end{array},$$

where feedforward *T*_*ff*_ and recurrent period *T*_*rec*_ are integrated together at membrane potential $V_{i}\left(t\right)=V_{i}^{f}\left(t\right)+V_{i}^{r}\left(t\right)$ and firing flag *S*=*S*^*f*^ + *S*^*r*^. The $V_{i}^{r}\left(t\right)$ saves the historical membrane potentials of the adjacent neurons. Furthermore, the recurrent SNN is designed with network dynamics from different scales, as shown in Figure 2A, where sparse or dense connections are given to the neurons in the same hidden layer.

### 4.3. Global Reward Propagation

Different from standard target propagations (a detailed description is shown in section 3.3), the reward propagation uses labels instead of errors as the teaching signals for the tuning of synaptic weights in the hidden layers, as shown in Figures 2A,H.

The reward propagation has been reported in our previous work, where only feedforward connections are introduced (Zhang et al., 2020b). Here, we update it to satisfy the criteria of both feedforward and recurrent propagations in the NRR-SNN architecture. The main idea is also trying to obtain the state differences from the propagated target gradient, which is defined as follows:

(7) $$\{\begin{array}{l} Grad_{RP}=B_{rand}^{f,l}*RP_{t}-h^{f,l}\\ \Delta W_{t}^{f,l}=-\eta^{f}(Grad_{RP})\\ \Delta W_{t}^{r,l}=-\eta^{r}\left(Grad_{t+1}+Grad_{RP}\right) \end{array},$$

where *Grad*_*RP*_ is the gradient of reward propagation, $B_{rand}^{f,l}$ is a predefined random matrix for the dimension conversion from the output layer to the hidden layer *l*, *h*^*f, l*^ is the current layer state, *RP*_*t*_ is the spike train repeated with one-hot labels, *W*^*f, l*^ is the synaptic weight at the feedforward procedure of the layer *l*, $W_{t}^{r,l}$ is the recurrent synaptic weight at layer *l*, *Grad*_*t*+1_ is the gradient calculated from the time *t* + 1.

### 4.4. Local Gradient Propagation With Pseudo-BP

Here, we use pseudo-BP to make the membrane potential differentiable, especially for those at the firing time. During the process of the torch.autograd in toolbox PyTorch, we set a “functional hook,” to store the spike signals and synaptic weight values generated from the feedforward procedure. This hook will then be automatically triggered as a backpropagate function for the pseudo-BP approximation in the feedback procedure.

The *Grad*_*local*_ is used to represent the local gradient from hidden membrane potentials to synaptic weights. In this procedure, the non-differential part is only the period of *V*_*i*_(*t*) at *V*_*i*_(*t*)=*V*_*th*_. Hence, the *Grad*_*local*_ of the neuron *i* is revised as follows:

(8) $$Grad_{local}=\frac{\partial S_{i}(t)}{\partial V_{i}(t)}=\{\begin{array}{cc} 1 & if\left(\left|V_{i}(t)-V_{th}\right|<V_{window}\right)\\ 0 & \text{ else} \end{array},$$

where only the differential parts are calculated or are otherwise omitted. The weight gradient of the full connection and loop connection will then be calculated by the automatic derivation mechanism of PyTorch.

### 4.5. The Learning Procedure of NRR-SNN

After integrating these three main parts, i.e., the neuronal plasticity, recurrent architecture, and reward propagation, we will get the integrated NRR-SNN.

The feedforward and feed-back propagations are shown in Figures 2B,D, where the *S*_*i,t*_ and *S*_*j,t*_ are the neuron-firing states, *V*_*i,t*_ and *V*_*j,t*_ are the membrane potentials, and *a* is the neuronal plasticity with adaptive threshold. This model has two time scales, containing *T*_*ff*_ for the feedforward propagation and *T*_*rec*_ for the recurrent propagation. The feedforward propagation connects a neuron's state at spatial scales, while the recurrent propagation links them at temporal scales. The neuronal plasticity has played important roles on the dynamic information propagation from the previous spike *S*_*i,t*−1_ to the next-step membrane potential *V*_*i,t*_ in the feedforward procedure in Figure 2B, and also the gradient propagation from *V*_*i,t*_ back to *S*_*i,t*−1_ in the feedback procedure in Figure 2D.

The vector field of the simplified dynamic LIF neuron is shown in Figures 2F,G, where Figure 2F shows an attractor at (1, 0), which means membrane potentials would move toward this stable point no matter where the initial point was, Figure 2G shows a saddle point at (−1, 0), which means that the point on the plane would move toward this point on one direction, but keep away from this point on another direction. The trend of these two directions would influence the other points on the plain.

An example of the relationship between neuronal plasticity with dynamic thresholds and spike trains is shown in Figures 2C,E, including the neuronal dynamics during learning the TIDigits dataset. The blue bar represents the sum of the *S*^*f*^ and *S*^*r*^. The *S*^*f*^ means the neuron firing state on the feedforward propagation and the *S*^*r*^ means the neuron firing state on the recurrent propagation. Therefore, the (*S*=*S*^*f*^ + *S*^*r*^) ∈ {0, 1, 2}. When the dynamic adaptive threshold *a*(*t*) < *Spike*, it would likely increase. When *a*(*t*) > *S*, it would have a negative attractor that could cause a decrease of *a*(*t*). The dynamic adaptive thresholds of different neurons would contribute to the feature learning during training, which would be further introduced in the following experiments.

The encoding of the NRR-SNN contains two parts: the network-input part and the inner-network part. For the first part, to retain the original data information as much as possible, we only resize the spectrum data by a scalar variable and then feed it directly into the network. For the second part, we encode information as the spike by comparing the signal to a threshold *V*_*th*_, where the signal above the threshold is set as 1, or else 0.

## 5. Experiments

### 5.1. Dataset Introduction

The TIDigits (Leonard and Doddington, 1993) and TIMIT (Garofolo, 1993) were selected as the two benchmark datasets for their sequential characteristics. The TIDigits dataset contains 4,144 spoken digits from zero to nine. Each sample in it was sampled as 20K Hz during 1 s and processed after fast Fourier transform (FFT) with 39 frames and 39 bands. TIMIT contains 630 American speakers, with 10 sentences for each person. Each sample was sampled as 16K Hz and processed after MFCC (short for Mel frequency cepstrum coefficient) with different frames and 39 bands. The frames were changed according to voice length, and the maximum was 780 frames.

**Figure 3:**
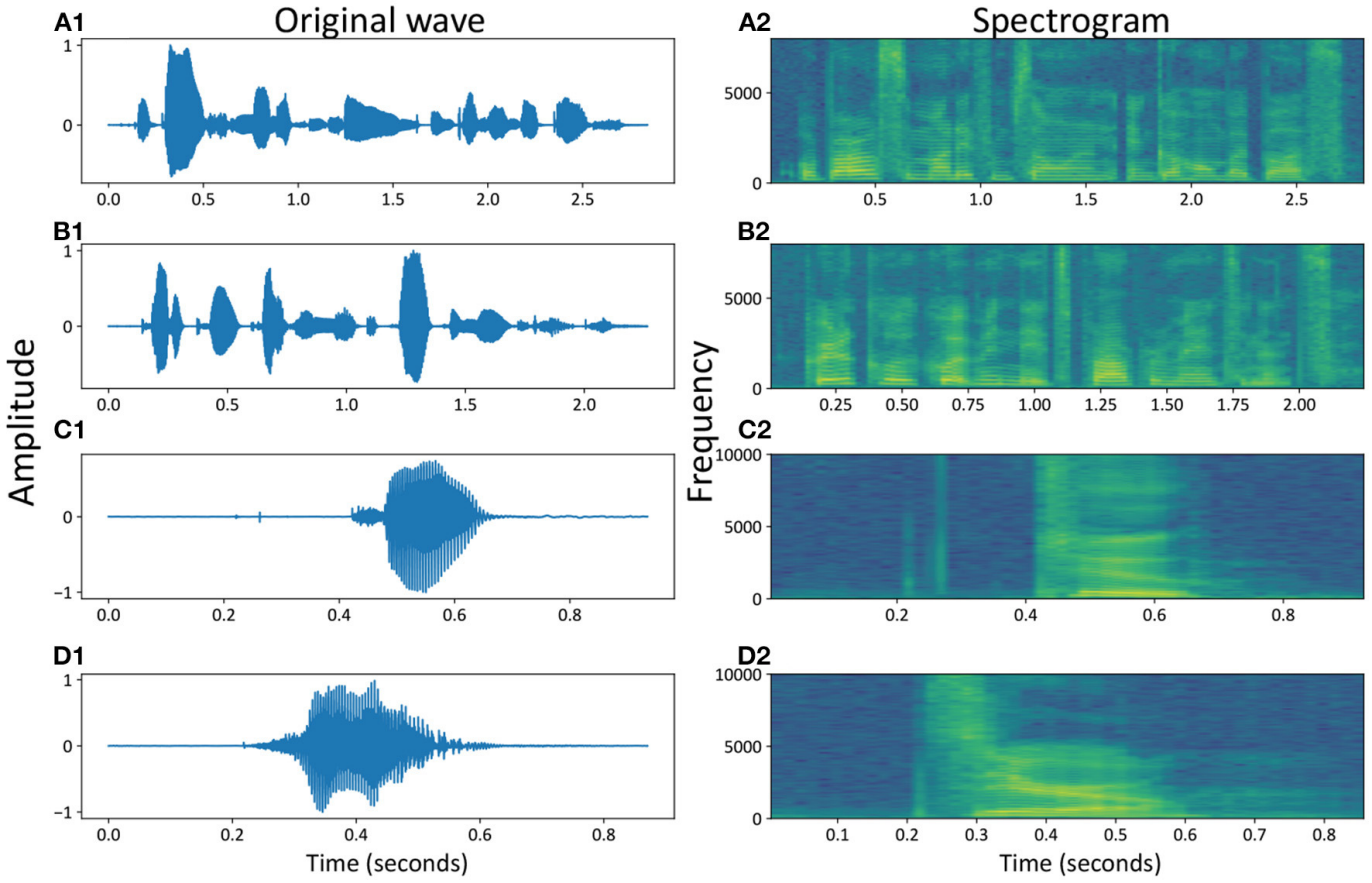
Speech waveforms and spectrograms of some samples, e.g., the temporal and spatial representations of spoken numbers, for example, “Or borrow some money from someone and go home by bus?” **(A1,A2)**, “Critical equipment needs proper maintenance.” **(B1,B2)**, “Two” **(C1,C2)** and “Zero” **(D1,D2)**.

For an easier description of the two benchmark datasets, Figure 3 shows the speech waveform of some selected samples, including the spoken word waves from the TIMIT dataset in Figures 3A1,B1 and the spoken numbers from the TIDigits dataset in Figures 3C1,D1. The waveforms of speeches were in line with our intuition, where the amplitude of the voice waveform would increase for voice signals. However, it was not easy to extract all of the high-dimensional information from the original waves directly.

In the time domain, the speech waves were converted into the frequency domain, called the speech frequency spectrum, to obtain more valuable speech information at high dimensions. Figures 3A2–D2 show four spectrograms of the same examples from the original waves in Figures 3A1–D1, respectively.

These two types of datasets covered most of the commonly used spoken words and numbers. From the temporal waves, we could find out that the spoken speeches in Figures 3A1,B1 were more complicated than spoken numbers in Figures 3C1,D1. Similar conclusions could also be found out from the spatial spectrograms, where more dynamics occurred in different voice bands of spoken speeches (with sentences) than spoken numbers (with simple words or numbers), with the MFCC parameters (Maesa et al., 2012).

In our experiments, the accuracy of TIDigits is defined as the number of correct identifying samples divided by the number of all samples. In contrast, the accuracy of TIMIT is defined as the number of correct identifying phonemes divided by the number of all phonemes, for the consideration of the multiphonemes in the same sample.

### 5.2. Parameters of the NRR-SNN

The key parameters of NRR-SNN for different tasks are shown in Table 1 from the scale of dynamic neurons to networks. In the table, *g* is conductance, *V*_*th*_ is the firing threshold of neurons, *τ*_*ref*_ is the refractory period, and *T* is the time window for the simulation of dynamic neurons. Furthermore, the capacitance of membrane potential was *C*=1*μF*/*cm*^2^, the reset value of membrane potential was *V*_*reset*_=0*mV*. For the reward propagation network, the loss function was selected as the mean square error (MSE), the optimizer was Adam, and the batch size was set as 50.

**Table 1:** NRR-SNN parameters for the two benchmark temporal tasks, where “RFC” is short for recurrent feedforward connection, and “FC” is short for feedforward connection.

**Tasks**	**Topology**	**Learning rate**	***g***	***V*_th_**	***τ*_ref_**	***T***	***V*_window_**
TIMIT	RFC500-FC10	Step wise from 1e-3	0.2 nS	0.5 mV	1 ms	10–100 ms	0.5 mV
TIDigits	RFC500-FC39	1e-4					

### 5.3. Neuronal Plasticity With Adaptive Threshold

**Figure 4:**
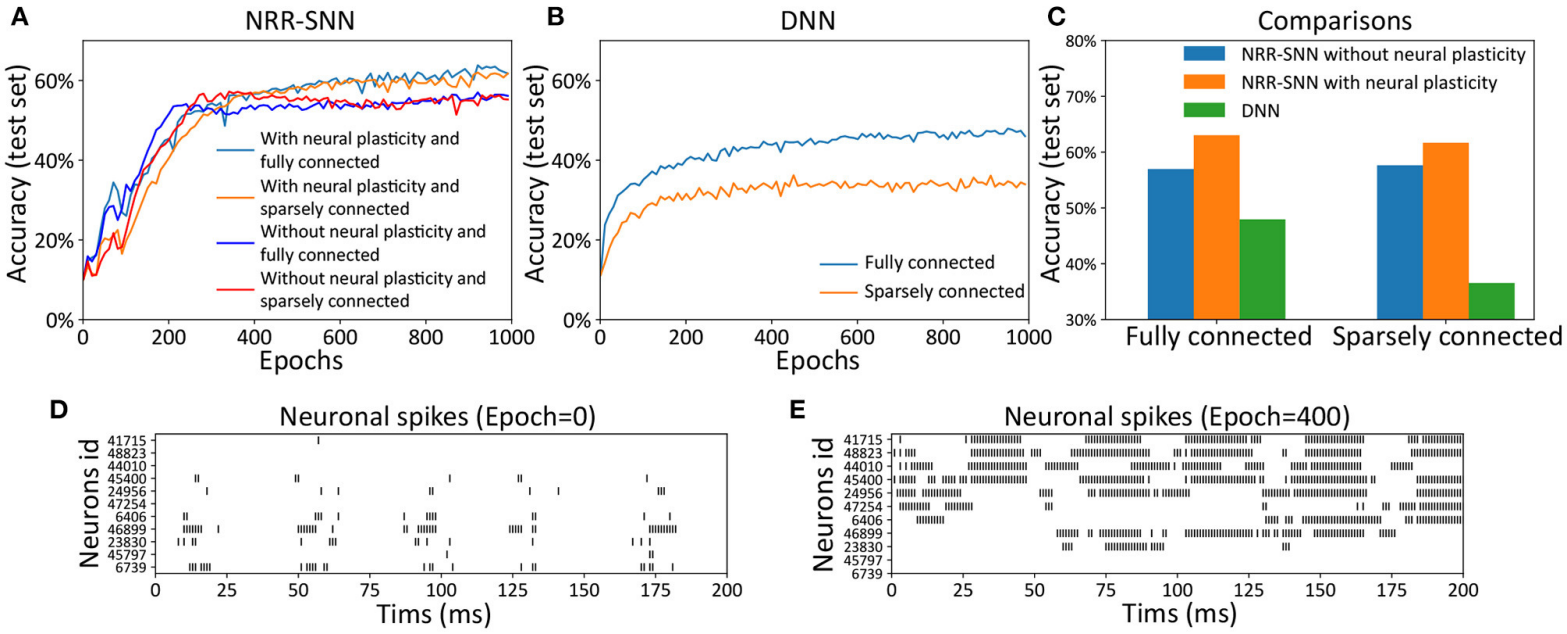
The neuronal plasticity and sparse connection for improving network learning. **(A)** The test accuracy of NRR-SNN with or without neural plasticity. **(B)** The performances of DNNs. **(C)** The performance comparisons of NRR-SNN and DNN, containing 100% connections and 50% sparse connections, with or without neural plasticity. **(D,E)** The neuronal spikes at different learning epochs, from epoch 1 to epoch 400.

We tested the NRR-SNN and DNN together, with or without neuronal plasticity (and 50% uniformly-distributed random connections), to better analyze the contribution of neural plasticity to the network learning. The results are shown in Figure 4, where the neuronal plasticity has played a more important role in improving test performance than that in BP based recurrent SNN (BP-RSNN).

The network of NRR-SNN with 50% sparseness connections had similar performance compared with that with 100% connections in Figure 4A. In other words, the sparse connections of neurons reduced power consumption without compromising performance. Figure 4B shows that the sparse connections could largely reduce the accuracy of speech recognition of the DNNs. Furthermore, Figure 4C shows that networks using neuronal plasticity could largely increase the test's accuracy. Considering that it took energy to pass information between neurons, the network's full connection would consume more computational resources during training. Therefore, the sparse connections of neurons would result in less consumption of computational cost.

Another hypothesis was that the sparse connections between neurons would decrease the network's complexity, but on the contrary, the additional adaptive threshold method of neurons would increase neurons' complexity. NRR-SNN was staying at a proper complexity for the efficient processing of information. This characteristic showed a good balance between neuronal complexity and network complexity.

During training, we also recorded the firing states of different dynamic neurons. Figures 4D,E show the neuron firing states from the beginning of training (e.g., epoch = 1) to the end of the learning (e.g., epoch = 400). For each epoch, the duration of signal propagation is 200 *ms*. Some neurons randomly selected from the NRR-SNN network are shown in the figure with the x-coordinate as the simulation time (*ms*) and the y-coordinate as the neuron index (id). The spikes for most neurons were sparser, and the spike count or fire rate was smaller at the beginning of learning (epoch = 1) compared to that at the end of learning (epoch = 400). Neurons also reached stable learning states with obvious periodic firing. Besides, some neurons had more confidence for the judgment of firing (e.g., the neuron with id 41715) by responding more strongly and quickly to the input stimulus, while some other neurons were tuned to have a weaker response to the same input (e.g., the neuron with id 6739).

### 5.4. Reward Propagation Contributed to the Neuronal Dynamics

**Figure 5:**
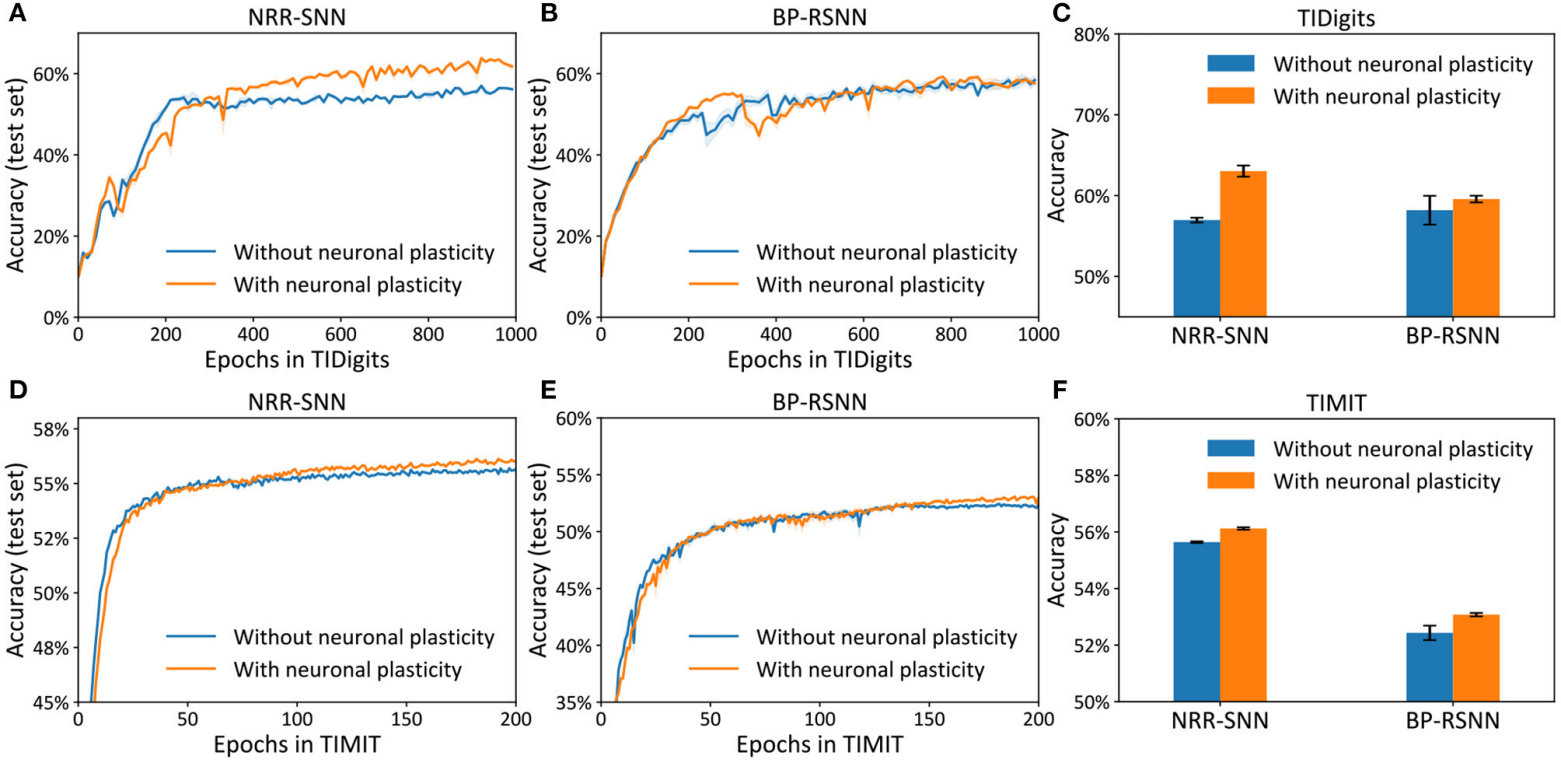
Accuracy on the test set for the models (i.e., NRR-SNN and BP-SNN) that with or without neuronal plasticity. **(A–C)** The performance on the two models on the TIDigits dataset. The test set for the models **(D–F)**, the performance on the two models on the TIMIT dataset.

The differences between the NRR-SNN and BP-RSNN (recurrent SNN trained with pseudo-BP) were with or without reward propagations. The proposed NRR-SNNs were convergent during the training of TIDigits in Figure 5A and TIMIT in Figure 5D. Besides, the models with adaptive thresholds showed higher test accuracies. The standard BP-RSNN models were also tested on these two benchmark datasets in Figures 5B,E and showed a smaller difference between those with or without neuronal plasticity. This result shows that NRR-SNN architecture could cooperate better with neuronal plasticity to some extent. Figure 5C showed the maximal test accuracies on the TIDigits dataset. The NRR-SNN and BP-RSNN reached 56.96 and 58.19%, respectively, without neuronal plasticity. After neuronal plasticity, the performance of NRR-SNN was increased to 63.03%, which was higher than BP-RSNN with 59.57%. A similar higher performance of NRR-SNN was also reached with the TIMIT dataset in Figure 5F, where NRR-SNN reached 56.12% accuracy and BP-SNN reached only 53.08% accuracy with neuronal plasticity. These experimental results showed that reward propagation contributed to the neuronal plasticity toward the higher SNNs' performance.

### 5.5. Robust Computation With Sparse and Recurrent Connections

The NRR-SNN contained tunable recurrent connections in the inner hidden layers that would contribute to the recognition performance, especially for the samples with noise (uniformly-distributed random noise).

**Figure 6:**
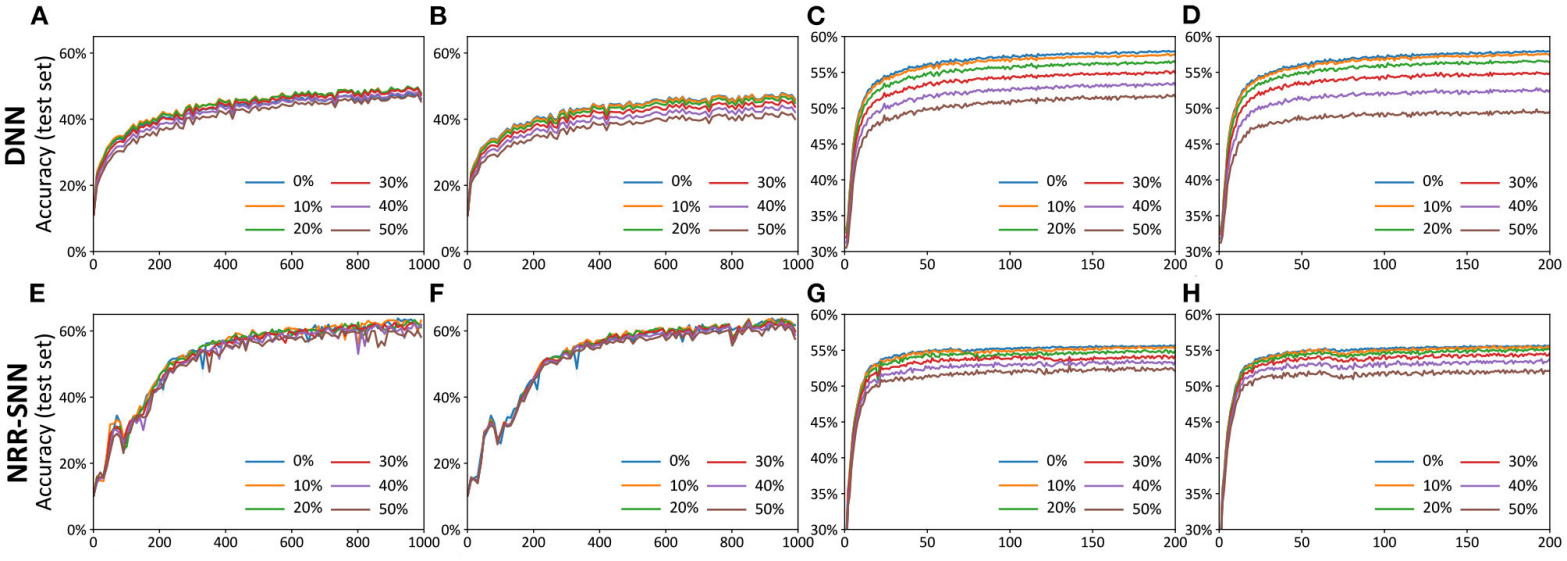
The comparisons of DNNs and NRR-SNNs for the robust computation on the samples containing noises. The “noise-noise” means that we added the noise both into the training dataset and test dataset. The noiseless-noise meant that we only added the noise to the test dataset without giving that to the training dataset. **(A)** Epochs on TIDigital noise-noise. **(B)** Epochs on TIDigital noiseless-noise. **(C)** Epochs on TIMIT noise-noise. **(D)** Epochs on TIMIT noiseless-noise. **(E)** Epochs on TIDigital noise-noise. **(F)** Epochs on TIDigital noiseless-noise. **(G)** Epochs on TIMIT noise-noise. **(H)** Epochs on TIMIT noiseless-noise.

Figures 6A–D showed the test accuracy of traditional DNNs, where the performances decayed quickly with the increase in the proportion of the noise. Unlike DNNs, the NRR-SNNs performed better toward the robust computation, where the performances were not changed as much with different proportions of noises on the TIDigits dataset and were only a slightly effected for those on the TIMIT dataset, as shown in Figures 6E–H. Obviously, the recurrent connections in SNNs were the key to keeping a robust classification of sequential information.

Furthermore, we used another standard indicator called accuracy-noise ratio to describe the performances of the robust computation, represented as $RobustRatio=\frac{Acc_{noiseless,noise}}{Acc_{noise,noise}}$, where *Acc*_*noiseless, noise*_ meaned “accuracy of noiseless data set for train and noise data set for test,” and *Acc*_*noise, noise*_ meaned “accuracy of noise data set for train and noise data set for test.”

**Figure 7:**
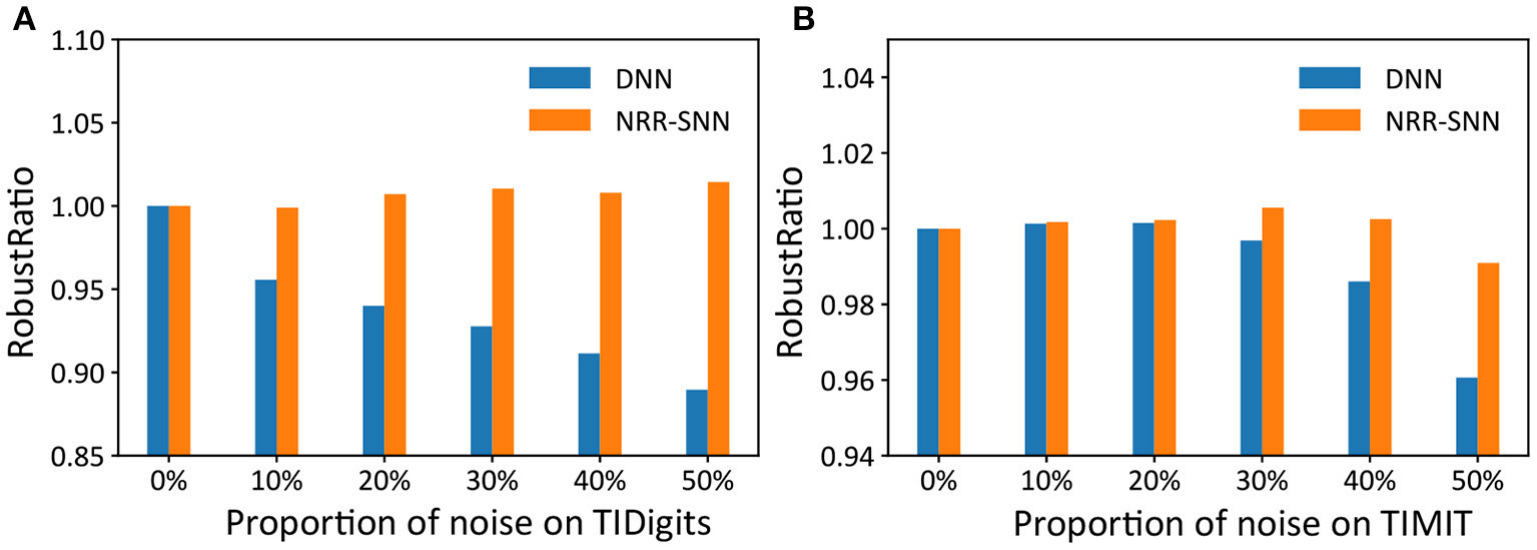
The comparison of robust ratios between DNNs and NRR-SNNs. The robust ratios of NRR-SNN decrease slowly compared to that of DNN on both sequential TIDigits **(A)** and TIMIT datasets **(B)**.

The performance of the robust ratio is shown in Figure 7, where even for the models trained with noise-free training data, accuracy was maintained when the noise ratio of the test data reached 50%. While for DNNs, they were sensitive to noise, and their recognition accuracy was significantly reduced with the increase of noise proportions.

### 5.6. The Comparison of NRR-SNN With Other SNN Models

In Table 2, we compared the performance of our NRR-SNNs (with bold marker) with other SNNs. An ablation study was further given, especially on the adaptive threshold, sparse loop, reward propagation, and shallow or deep architectures on SNNs.

**Table 2:** The performance comparison of our NRR-SNN model with other spiking models.

**Task**	**Architecture**	**Training type**	**Learning rule**	**Performance** ** (%)**
TIDigits	SOM-SNN (Wu et al., 2018)	Rate+Spike	SOM+BP	97.40
	Liquid state machine(Zhang et al., 2015)	Spike-based	BP	92.30
	**Pure feedforward SNN**	**Spike-based**	**Pseudo-BP**	**36.25**
	**Feedforward with adaptive threshold**	**Spike-based**	**Pseudo-BP**	**66.05**
	**Feedforward with sparse loop**	**Spike-based**	**Pseudo-BP**	**60.86**
	**Shallow NRR-SNN (three layers)**	**Spike-based**	**RP**	63.03
	**Deep NRR-SNN (five layers)**	**Spike-based**	**RP**	**98.34**
TIMIT	Recurrent-SNN (Bellec et al., 2020; Wu et al., 2020b)	Spike-based	BPTT	73.60
	LSNN (Bellec et al., 2020)	Spike-based	E-prop	65.40
	**Pure feedforwardSNN**	**Spike-based**	**Pseudo-BP**	**52.97**
	**Feedforward with adaptive threshold**	**Spike-based**	**Pseudo-BP**	**53.42**
	**Feedforward with sparse loop**	**Spike-based**	**Pseudo-BP**	**55.67**
	**NRR-SNN**	**Spike-based**	**RP**	**56.12**
	**BP-SNN**	**Spike-based**	**RP**	**53.08**

*The bold values were the experimental results reached by different subtypes of the proposed NRR-SNN*.

It was obvious that our NRR-SNN reached the best performance on the TIDigits dataset. The pure feedforward SNN with three layers reached 36.25% tuned with Pseudo-BP. Then SNN with an additional adaptive threshold reached 66.05% accuracy, while those with additional sparse loops reached 60.86% accuracy. We also tested NRR-SNN with different configurations. The NRR-SNN with shallow architecture (three layers, with only one feedforward hidden layer with recurrent loops) obtained 63.03% accuracy, while a deeper one (five layers, containing input layer, convolution layer, feedforward layer with recurrent loops, feedforward layer, and output layer) obtained 97.40% accuracy, higher than some other SNNs, such as those based on the self-organizing map (SOM) (Wu et al., 2018) or liquid state machine (LSM) (Zhang et al., 2015).

For the TIMIT dataset, the shallow feedforward SNN reached 52.97% by Pseudo-BP. Then accuracies increased to 53.42% after adding the adaptive threshold and to 55.67% after adding sparse loops. It was reported that the accuracy of SNNs reached 73.06% for those with recurrent connections (RSNN) (Bellec et al., 2020; Wu et al., 2020b), and 65.40% for those with LSTM-based (long short-term memory) spiking neural networks (LSNN) (Bellec et al., 2020).

We established that our NRR-SNN reached 56.12%, which was lower than the previous RSNN and LSNN. However, we also noticed that the accuracy of NRR-SNN was still higher after replacing RP with BP (only 53.08%). We thought this was already a good indicator to show the performance of NRR-SNN, since the lower accuracy compared to other SOTA methods was more than the different sample lengths of TIMIT, where all of the samples in the same patch were normalized as the same length by padding zero to short samples. However, these problems are currently not in the scope of this paper.

## 6. Conclusion

Most of the research related to SNNs focuses on synaptic plasticity, including the STDP, STP, and other biologically-inspired plasticity rules. However, inner neurons' plasticity also plays important roles in the neural network dynamics but is seldom introduced. This paper's important motivation is to improve the performance of SNNs toward higher classification accuracy and more robust computation for processing temporal information with noises. A special Neuronal-plasticity and Reward-propagation improved Recurrent SNN (NRR-SNN) have been proposed for reaching these goals:

- The historically-related adaptive threshold with two channels is highlighted as important neuronal plasticity for increasing the neuronal dynamics.
- Instead of errors, global labels are used as a reward for the paralleling gradient propagation.
- Dynamic neurons are then connected in a recurrent loop with proper sparseness for the robust computation.

The experimental results have shown the proposed NRR-SNN's efficiency compared to the standard DNNs and other SNNs.

## Data Availability Statement

The TIMIT dataset can be downloaded from: https://catalog.ldc.upenn.edu/LDC93S1. The TIDigits dataset can be downloaded from: https://catalog.ldc.upenn.edu/LDC93S10.

## Author Contributions

TZ and BX conceived the study idea. TZ, SJ, XC, and HL conducted the mathematical analyses and experiments and wrote the paper together. All authors contributed to the article and approved the submitted version.

## Conflict of Interest

The authors declare that the research was conducted in the absence of any commercial or financial relationships that could be construed as a potential conflict of interest.
